# A gutsy approach to stem cells and signalling: an interview with Hans Clevers

**DOI:** 10.1242/dmm.013367

**Published:** 2013-09

## Abstract

Hans Clevers, Professor of Molecular Genetics at Utrecht University, began his career in immunology and developmental biology, but a shift towards intestinal research in the late 1990s led to his group’s pioneering discovery that Lgr5 is a marker of tissue stem cells – a finding that paved the way for a cascade of key insights into the molecular signalling pathways that are dysregulated in cancer. Interviewed here by Ross Cagan, Editor-in-Chief of *Disease Models & Mechanisms*, Hans recalls the mentors and discoveries that motivated his transition from basic to applied science, discusses his style of lab management and mentorship, and highlights the potential of organoid-based therapy for personalised medicine.

Johannes (Hans) Clevers was born in 1957 in Eindhoven, home to Philips Electronics, in the south of The Netherlands. From a young age he showed enthusiasm and a natural talent for science, and as an undergraduate became fascinated with molecular biology. He obtained his PhD in immunology from Utrecht University during the mid-1980s, and simultaneously studied medicine. Making the pivotal decision to move back into the lab after completing his clinical training, he undertook postdoctoral research in Cox Terhorst’s lab at the Dana-Farber Cancer Institute at Harvard University. He then returned to Utrecht to set up his own lab, and was a Professor of Immunology at the university between 1991 and 2002. From 2002 to 2012 he was Director of the nearby Hubrecht Institute for Stem Cell Research. During this time, Hans moved gradually into the gastroenterology field, and made groundbreaking discoveries regarding the role of Wnt signalling in stem cells and colon cancer. His unique contributions to cancer, stem cell research and regenerative medicine have been recognised in the form of numerous awards, and in 2013 he was one of the eleven winners of a $3 million award from the Breakthrough Prize in Life Sciences Foundation. Currently, he is Professor of Molecular Genetics at Utrecht University, and is also President of the Royal Netherlands Academy of Arts and Sciences (KNAW). Hans has also been involved in setting up several biotechnology companies.

**Before we get to your background, I want to congratulate you on being, unsurprisingly, one of the Breakthrough Prize award winners. You have a long list of prizes now – is it something you’ve gotten used to?**

This last one was unusual for me – prior to the Breakthrough award I had only ever received one American prize and that was in gastroenterology. To be the only researcher in Europe awarded, and to see my name on the list together with people like Robert Weinberg and Bert Vogelstein, who were the big shots when I was a postdoc, was a truly great honour. I went to the ceremony for the physics prize in Geneva, and it was like being at the Oscars – very surreal, as a scientist.

The first thing I did when I found out about my award was to invite the current and previous members of my lab to a huge party in Amsterdam, which will take place in September [2013]. There will be around 100 attendees – most of which are still in science. There will be good food and drink, stand-up comedy, and a small symposium.

**Figure:**
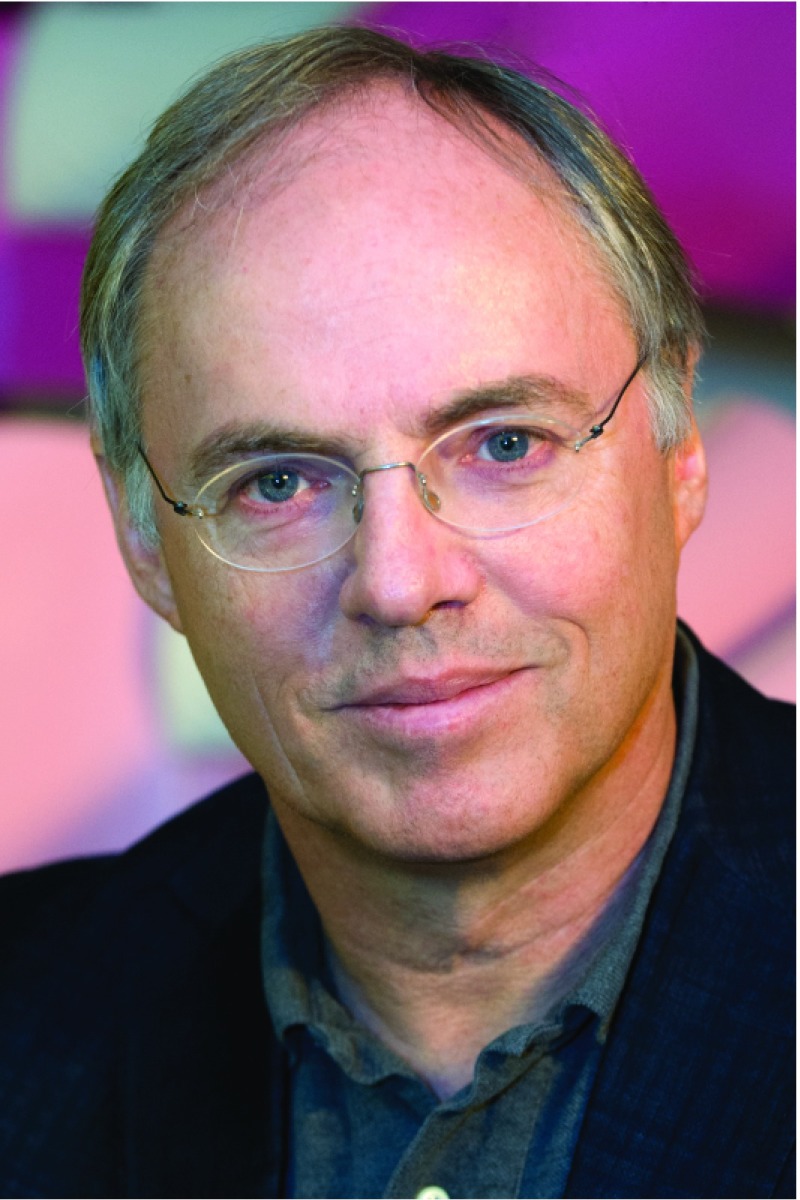


**Taking a step back into your past, why did you choose a career in science and medicine?**

My high school system was very geared towards languages. I started learning biology at university in 1975 at the age of 18, and I was disappointed. Molecular biology was being developed in England, Switzerland and the US, but in Dutch universities there was no legal framework to do this, and so the courses – where available – focused only on technical details. Biology in general lacked charisma. At the time, my friends and brothers were junior medics, and as I had an interest in medicine I decided to take it on in addition to biology. I ended up spending a year in Nairobi and half a year at NIH for my biology rotations, and essentially I never went to any lectures (although this is something I never tell my students!). Anyway, I really started getting sucked into the clinical training, and found that working in a clinical environment is much more sociable than being in a lab. You’re part of a big organisation and there are lots of people to talk to, whereas in the lab there are only a few people, and small issues – such as somebody not cleaning up – can really cause friction. After medical school, I was picked, mainly because of my research background, for a training position in paediatrics. They suggested that I should start work for a PhD, so I went back into the lab. That’s when I realised that, despite the social attractiveness of working in a hospital, I was much more of a scientist than a doctor. I got my PhD – together with four published papers – in just 1 year. However, it was during my first postdoc position in Boston that I think I was really exposed to science for the first time. It was tough, but I knew I’d made the right decision.

**Are there particular mentors who influenced your decision to choose the lab over clinics, and shaped your career moves?**

When I received the Heineken Prize from the Royal Netherlands Academy of Arts and Sciences in 2012, I had to think deeply about my mentors and realised that there were two that I had almost forgotten. The first was my high school chemistry teacher, who sold laboratory chemicals to students from his home, during the evenings (in a well-regulated way). I had built a small lab in the attic of my parents’ house and I really had fun mixing things together and doing all the experiments that are possible to do at home. Because of this chemistry teacher, I learned the joy of being in a lab.

The second crucial mentor was my thesis advisor, who didn’t supervise me very much but did give me key advice that has stayed with me until now. He taught me that it’s important to trust everybody you work with, at least until they show you that they can’t be trusted. I emphasize this in my own lab – I encourage my students and postdocs to be open and transparent and to discuss their work. Some scientists are intuitively secretive and paranoid – cultural differences perhaps play a part in this. In my view, only when someone damages your trust can you justify being paranoid, and until then it is important to share information.

“…it’s important to trust everybody you work with, at least until they show you that they can’t be trusted”

**There are many ways to run a lab; for example, you can micro-manage it or you can focus on the big picture and step back from the day-to-day issues. What is your style of running a lab?**

When I first became a PI, I really liked doing experimental work. Even after 5 years as a postdoc, I enjoyed doing minipreps! As a consequence, I really micro-managed the few lab members I had, and I’m sure they were ultimately happy to get away from me. But when the lab grew a little bigger and I became Head of Department, it took me away from the lab much of the time. Nowadays, I informally talk with my lab colleagues as much as I can, preferably at the bench. As we speak, I know that there is someone in my group who will find out the results of a 3-month effort, today. I always insist on looking at the raw data, never the digested, analysed data. It could be 5 minutes or 2 hours, but when I’m needed in the lab I will always try to make time for it and be part of the troubleshooting process. When you can no longer troubleshoot in your own lab, you’re lost.

**Well clearly success builds on success – some impressive scientists have come out of your lab. Do you encourage all of your group members to pursue academic positions?**

I’ve had many ‘super postdocs’ in my lab but some of these individuals would not be happy as PIs. It’s not about capability, but about wanting to deal with the paperwork, the responsibility and the decision-making that come with being a PI. Such individuals can make a valuable contribution to a lab, given their years of experience, as well as acting as great mentors and role models for the newer group members. When, having gained experience in the pharmaceutical industry, Nick Barker re-joined my group in 2006 as Senior Staff Scientist, we spent 6–7 years looking for stem cell markers, and then broke open the field by identifying Lgr5 as a marker of cancer stem cell populations. Nick has now set up his own group in Singapore, but I have had several other very talented experimentalists in my lab for many years. Overall, I think that intermediate positions are fantastic for successful postdocs who might end up unhappy as PIs.

**How did you get involved with intestinal stem cell research? You didn’t start in this field but somehow ended up there.**

As an undergraduate student, I did a brief rotation project on T cells. This led to a PhD and postdoc focused on T cells. I learned molecular biology, which inspired me to clone a T-lymphocyte transcription factor, TCF-1, when I subsequently set up my own lab in Holland. We (Marc van der Wetering and I) cloned TCF-1 within a few months and showed that it binds DNA; but, despite trying all kinds of functional assays, we couldn’t show that it regulates transcription. It took 6 or 7 years to figure out that β-catenin, a signal transducer in the Wnt signalling pathway, was needed. We heard that Walter Birchmeier had made a complementary discovery in Berlin, and our papers came out at the same time.

Around that time, I was Clinical Professor in Immunology at Utrecht, and I started studying TCFs in mice, frogs, flies and worms. We soon established that TCFs are always the endpoint of the Wnt pathway. In 1996–1997, we knocked out TCF-4 in mice and, remarkably, observed a gut phenotype – the mice had no crypts. Simultaneously, we realised that the pathway is overactivated in colon cancer. That’s when I decided to move into studying the gut. It wasn’t easy as an immunologist, but I gradually got to know the gastroenterology field. At the time, this field was dominated by clinical research, and in fact our work didn’t really become known to gastroenterologists until around 3–4 years ago. They were totally unaware that mice could give clues about human disease, which surprised me, as in haematology and immunology, there is a good balance between basic and clinical science. There are other clinically well-developed fields, such as prostate and lung cancer research, that could really benefit from a stronger basic approach.

**A key discovery for you was that Lgr5 is a marker of stem cells. When did you realise the implications of this discovery?**

There were two ‘eureka’ moments with the stem cell story. The dogma at the time was the ‘+4’ stem cell model, which was pioneered by Chris Potten, who recently passed away. I tried to provide experimental support for this model, together with Nick Barker, but it never really went anywhere. Having realised that β-catenin and TCFs controlled crypts in the gut and cancer, we set out to determine the genetic programme controlled by this pathway. At the time (1997), there was no technology to do this properly, but in 2000 we performed one of the first microarrays with Pat Brown. Our array looked at expression in a colon cancer cell line. The array contained only two samples – plus or minus the Wnt pathway – but it opened the field for us by providing a list of markers to investigate further. This was the first, key step. From the list of markers, we picked a few that we thought were marking +4 cells, but these led us nowhere. Eventually, based on its unique expression pattern, we came up with Lgr5. We made numerous mouse strains, including Lgr5-GFP tagged mice. The moment we saw tiny cells lighting up under the microscope, I started writing our next ten big papers in my head. It was a remarkable moment – the cells exist, and we could visualise them using these mice.

**And why exactly is Lgr5 so important, both from a basic and an applied standpoint?**

Lgr5 is an exquisite protein. We and several other labs have shown that it is a marker for stem cells in many tissues. Originally, we saw it only in spontaneously dividing tissues, but we’ve recently found that it also appears in organs that have undergone damage. Lgr5 is unique in that it – on its own – it specifically marks homogenous populations of stem cells but not their progenitors, unlike most other markers. We now know that this is because it is a cell surface receptor protein in the Wnt pathway, and only stem cells require Wnts. In the gut, the stem cells are particularly active – in mice, they divide every day for 2.5 years, so they go through a thousand cell divisions.

Discovering Lgr5 led to another eureka moment: the generation of long-term culture systems that maintain crypt physiology. A Japanese gastroenterologist who I invited to my lab, Toshiro Sato, was the first to set up the right culture conditions, and now multiple labs are creating these systems, which are called organoids or ‘mini-guts’. Once the system was up and running, Toshiro showed that Paneth cells provide the niche for stem cells at crypt bottoms, and that stem cells produce their own daughters which then produce growth factors. With his former Japanese lab, we showed that normal tissue can be generated from a single stem cell, and it can survive in a mouse for as long as you want. Based on this finding, our lab evolved and now we’re culturing prostate, liver, pancreas, kidney, lung and breast tissue, all for prolonged periods of time, all from humans. There are no changes in chromosomal structure in the cultured cells, and deep sequencing reveals very few mutations. The next step will be to take single cells, genetically modify them like we do with embryonic stem cells, pick a safe clone, expand it and use it for therapy, particularly transplantation.

**Do you think we will be able to take organoid-based therapy to the personalised level? Colorectal cancer, for example, only has a 3% success rate in clinical trials. Are organoids going to provide the answer?**

We’re finalising a pilot sequencing study now involving 20 patients with normal crypts and colon cancer. With the wild-type and colon cancer organoids, we can potentially predict patient outcome and response to drugs. In the future, we hope to rapidly build large, living biobanks for other cancers, too. In line with this, we’re building up a ‘Stand Up 2 Cancer’ dream team involving several American labs and the Sanger Institute, with the aim of taking the organoid approach to the next level in cancer therapy. Sanger has robotised screening set-ups that allow thousands of compounds to be screened across hundreds of cell lines. We can now do this with patient-derived organoids. From these tests we could establish new effective drug combinations, and we could link genetics to function to help design smarter trials. The great thing about organoids is that they contain only epithelium – there is no immune system, no blood system, only the diseased tissue, making it a very clean system.

We’ve also recently collaborated with clinicians on a cystic fibrosis project. We can predict using cystic fibrosis ‘mini-guts’ that certain drugs that are currently in trials will work for one patient and not for another, and that certain drug combinations work better than others. From biopsy to drug response, it takes only 10 days. Industry is now very interested in using this assay to pre-screen and design trials.

“The great thing about organoids is that they contain only epithelium – there is no immune system, no blood system, only the diseased tissue, making it a very clean system”

**In the past, you’ve suggested that classic hypothesis-driven science isn’t the right way to do science. Could you say a little bit more about this?**

Now that I’m a bit older I’m more interested in how the process of science works. I always ask my colleagues: how do you run the lab and how do you make discoveries? In my lab, I try to establish a reproducible, quantitative system, like GFP mice and arrays. Then, I throw something at the system and look, without formulating a hypothesis. This is difficult because our brains like to produce causal relationships, even though these are often wrong. I’m constantly telling my group members that they should keep their minds open and make observations without assuming that they know what’s going on. In molecular biology, we can go anywhere we want and there are billions of effects to discover. You cannot do this in a hypothesis-driven way because you’re essentially retracing evolution. There are many solutions to a particular problem but evolution picked one – it’s very arrogant to think we can reconstruct this in our minds.

**Some of my most elegant hypotheses have fallen by the wayside. The importance of establishing formal rules for innovation is a discussion worth having in biology. I understand that you have embraced movies to explain scientific concepts. What’s the story behind this?**

I was inspired by Leonard Zon – I came across one of his movies about 8 years ago. I realised it’s much easier to convey messages visually than in words so I started working with a small company in Holland to produce science movies. The lab provides the idea and the images, and the company writes the script. We end up going back and forth a few times to make the message as accurate as possible, and it really shows us as scientists how ambiguous language can be. Often, feedback from the company sends us back into the lab to find out something we hadn’t looked into, for example how fast do the cells move, how many cells are there? Gradually, the movie comes together. Nowadays, I typically use the movies in my talks to explain a problem, and I’ve found that it’s much more effective to show the movie before explaining the experiments. People understand the experiments much better that way, and listen effortlessly. Now, whenever we have a story to write up I try to turn it into a 30-second movie before putting pen to paper. This really forces us to think about the core of the paper.

“In molecular biology, we can go anywhere we want and there are billions of effects to discover…There are many solutions to a particular problem but evolution picked one – it’s very arrogant to think we can reconstruct this in our minds”

**In your view, is being a scientist a good career choice? What advice would you give to a young scientist thinking about this career?**

Science is frustrating because things don’t work 90% of the time: ideas are wrong, experiments fail. You have to have the personality that thrives by those few fantastic moments of success that you have once a year or even once a career. Moving from being a clinician to being a scientist was one of the hardest decisions I ever made. A clinician gets rewards multiple times a day, so if you’re a person who needs that kind of reward and social interaction, then you shouldn’t be a scientist. Luckily there are now many alternative careers, such as pharma, government and teaching, that didn’t exist when I was a young scientist. However, there needs to be a radical change in the way we view these alternative routes. Maybe in the US it’s different, but here, if you step out of the system you are treated like a failure. I tell young scientists that failure comes with ending up as a miserable PI, with no funding and no papers.

PhD students and junior postdocs have to be aware that the people they see at meetings who give the great talks are in the minority – as scientists we have to be ready to do something else at any point during our career. I think the whole system has to realise that every other job can be as interesting as a job in science. That’s not what we always convey to young people – we describe academia as where it’s happening and everything else as dull or uncreative.

**If you hadn’t chosen science as a career, what would you have done instead?**

I would probably be a novelist. It’s even more competitive than being a scientist, but it’s also creative, so the perfect blend for me.

**What do you do to relax away from the lab?**

I do a lot of sport, including running and skiing, with the kids if possible. I have completed the New York marathon several times. I also read several books a week.

